# ALFRED: An Allele Frequency Database for Microevolutionary Studies

**Published:** 2007-02-22

**Authors:** Haseena Rajeevan, Kei-Hoi Cheung, Rohit Gadagkar, Shannon Stein, Usha Soundararajan, Judith R Kidd, Andrew J Pakstis, Perry L Miller, Kenneth K Kidd

**Affiliations:** 1 Department of Genetics, Yale University School of Medicine, New Haven, CT 06520-8005, USA; 2 Center for Medical Informatics, Yale University School of Medicine, New Haven, CT 06520-8005, USA

**Keywords:** ALFRED, allele frequency, database, population sample, polymorphism

## Abstract

Many kinds of microevolutionary studies require data on multiple polymorphisms in multiple populations. Increasingly, and especially for human populations, multiple research groups collect relevant data and those data are dispersed widely in the literature. ALFRED has been designed to hold data from many sources and make them available over the web. Data are assembled from multiple sources, curated, and entered into the database. Multiple links to other resources are also established by the curators. A variety of search options are available and additional geographic based interfaces are being developed. The database can serve the human anthropologic genetic community by identifying what loci are already typed on many populations thereby helping to focus efforts on a common set of markers. The database can also serve as a model for databases handling similar DNA polymorphism data for other species.

## Introduction

Microevolution of populations or subspecies within species is frequently based on allele frequencies at multiple polymorphic genetic loci. Studies that examine evolutionary history of genes in humans and many other organisms have generated a wide variety of genetic variation data using different experimental methods to assay DNA variation including high throughput methods (eg, microarray methods (Zhong, 2003, Gunderson, 2005) and TaqMan-based SNP genotyping (Livak, 2003)). Databases play a crucial role in managing and querying, analyzing, and visualizing these data. ALFRED (http://alfred.med.yale.edu) is a database that stores allele frequency information on a large combination of DNA polymorphisms and human population samples (Osier et al, 2002). (“ALFRED” is an acronym for ALlele FREquency Database.) Though used for human data, much of its structure would apply to any species with distinct populations or samples from distinct geographic regions studied for polymorphisms of any sort, not just DNA based polymorphisms.

While many biologists tend to think of genes as functional units with fixed physical locations on the chromosome, population geneticists are more interested in studying the DNA sequence polymorphisms (which vary across individuals in a population) in a given chromosomal segment (eg, a gene). In our data model, we consider the individual specific polymorphisms as “sites” within a “locus” where “locus” can be a functional gene or an arbitrarily defined segment of DNA that may have no coding regions nearby. An allele at a site is a specific nucleotide sequence. Alleles can be of different lengths or just differ at a single nucleotide. Tables in ALFRED can handle all types of DNA polymorphisms including Single Nucleotide Polymorphisms (SNPs), insertion/deletions (indels), Short Tandem Repeat Polymorphisms (STRPs), Variable Number Tandem Repeats (VNTRs) and combinations of multiple sites (haplotypes). So that the same polymorphism can be studied by multiple laboratories and the results compared, it is essential that the polymorphism be described unambiguously. In ALFRED this involves not only storing relevant DNA sequence but also protocols and links to such international databases as dbSNP where the full genomic context is given.

As ALFRED is designed to address anthropological and evolutionary research questions, it includes the detailed description of the population samples (groups of individuals from whom DNA materials were collected). Such information includes the geographic context and language spoken, at a minimum. Because relevant research questions involve variation in allele frequencies, the samples of individuals used to calculate an allele frequency need to be considered separately from the populations they represent. Various ascertainment biases could exist and even a single population (defined, say, linguistically) can have genetic structure.

As of September 2005, ALFRED contained 35,531 frequency tables (a single population sample typed for a single site) for 1302 sites in 573 loci. There are >1382 population samples typed for at least one of these sites. Note that not all sites are typed for all populations and vice versa. The general emptiness of the site X population data matrix is a problem for the field, as discussed later.

## Database evolution

ALFRED has gone through different stages of development. In the initial stage, we used the rapid prototyping technique to identify the user needs and to design and develop the database system. To implement the prototype system, Microsoft’s Access (which is a PC-based relational database package) was used to create the database. Access is developer friendly in terms of its ease of code development and code change. Access provides an easy-to-use graphical interface for developers to create tables and forms for data queries and display. This was critical as the initial development of ALFRED was an interactive and iterative process in which system requirements tended to change rapidly. The Web front-end was built using Active Server Pages (ASP). Most of the user interface code was written in Visual Basic scripts (VBscripts) and database access was implemented using Open Database Connectivity (ODBC). We used Internet Information Server (IIS) as our Web server (ASP is a part of IIS), which runs on Windows 2000. The use of Access together with ASP enabled us to achieve rapid prototyping.

With the system becoming more stable and the size of the database growing, we began to look for a more robust and powerful database engine. We have migrated the ALFRED database from Access to Oracle (version 9i). The database as well as the user interface code required several modifications to ensure complete compatibility.

Daily curation of ALFRED data is performed through a separate web application that is password and firewall protected. This application is written in Java using servlets, JSPs, JDBC, Java Mail and HTML. In addition every curatorial action through the application is logged.

## Structure and data contents

**Figure 1:**
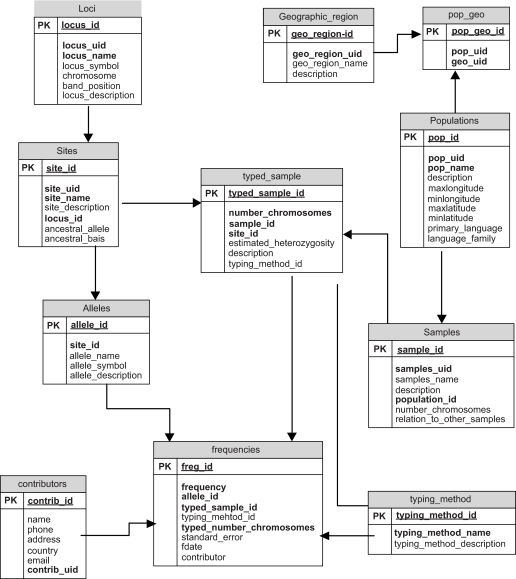
Core structure of ALFRED

ALFRED is a relational database, with the data stored in tables that are related through the primary-foreign key mechanism. The core structure of our database, as shown in Figure 1, consists of tables representing information on polymorphisms (**Sites** table) within a locus (**Loci** table) and samples (**Samples** table) of populations (**Populations** table). **Populations** are organized by geographic regions (**Geographic_Region** table). In our database representation, one population can have more than one sample. It is important to maintain distinction of separate samples of the same population (especially for such heterogeneous populations as African American and European American) collected from different geographic regions but grouped under the same population. Population samples are typed to determine frequencies of alleles (**Alleles** table) at a site. The linkage between a *polymorphism* and a *sample* is captured in the **Typed_Sample** bridging table. In addition, the **Typed_Sample** table associates the typing method with a specific frequency. Multiple different typing methods exist for characterizing many polymorphisms and could be developed for almost any polymorphism. Almost all methods can be affected by differences in the DNA sequence other than those occurring at the site being assayed, whether in the adjacent few nucleotides for 5′ exo- and oligo annealing assays or more distantly under the 3′ end of a PCR primer. Whether such additional variation is relevant to a given allele frequency will depend on the typing method used. The allele frequency data for each **Typed_Sample** entry are stored in the **Frequencies** table. The typing method used for typing the site for allele frequencies is stored in the **Typing_Method** table. Detailed descriptions of the individual tables (including their fields) are available via: http://alfred.med.yale.edu/alfred/table_list.asp.

All publication related information is stored in a single **Publications** table and intermediate tables are defined to link **Publications** to **Frequencies, Samples, Sites** and **Loci.** Links to other Web sites are stored in the **URLs** table. These links are associated with the **Loci, Sites, Populations** and **Publication** tables. All frequency records are linked to the contributor (**Contributors** table), which stores information about individuals who contribute the allele frequency data.

Every record in ALFRED has a unique identifier (UID). The UIDs are a text string consisting of three parts: a Table Identifier, a Record Number, and a Check Character. The Table Identifier is a two character symbol representing the table the record belongs to, such as PO for POpulations, LO for LOci and so on. The Record Number is an internal identifier for the specific record. The Check Character is a simple checksum for the digits in the Record Number. The Check Character is determined by summing the digits of the Record Number, taking the modulo 26 of that number, and representing the resulting number as an upper case ASCII character (A-Z). For example, LO000423J is the UID for the locus ADH4 (the prefex “LO” indicates the UID refers to a locus, the suffix J is the Check Character, and 000423 is a number generated by the system when the record is created). Based on these UIDs, we can create URLs in the following format: http://alfred.med.yale.edu/alfred/recordinfo.asp?UNI D=<*UID*> (where <*UID*> will be replaced by the actual UID value).

Other web resources can utilize these URLs to access ALFRED for allele frequency tables and related information. In addition, ALFRED UIDs are mapped to corresponding dbSNP accession number (rs) and GDB ids. This mapping information has allowed us, for example, to utilize the *LinkOut* function provided by NCBI’s search interface ‘Entrez SNP’. This makes ALFRED’s data (eg, polymorphism information and subsequently allele frequency distribution graphs and tables) accessible from NCBI.

ALFRED curators follow certain guidelines when extracting data from the literature. Locus/site information and population/sample information along with the actual data have to meet specific criteria. For example, some researchers publish data on “Asians”. However, the term “Asians” is so general and can refer to a diverse group of populations with genetic differences among them (Kim et al, 2005). If the curators can determine that it was a heterogenous sample, i.e., a mixture of individuals from different ethnic groups, the data do not meet our criteria and therefore would not be included in ALFRED. Alternatively, the curators may learn that the sample was ethnically homogenous and it would then be included under the correct ethnic/population label. Curators also regularly review data already in ALFRED to make sure that the allele frequency data and related information accessed by the users are up-to-date and error free. Descriptions for loci, polymorphisms, alleles, populations, and samples are added/updated and expanded on a daily basis. Web links to public databases such as GenBank, PubMed, GDB, OMIM, Entrez Gene and Ethnologue are added, as are relevant links to the literature. Ambiguities and blatant errors in allele frequency data from publications are rectified by communicating directly with the corresponding author of the related publication. For example, typographic errors can result in allele frequencies not summing to 1.0. Without checking with the original authors it is usually not obvious what the error is, only that there is an error.

While the basic structure of the database has not changed since the last paper (Rajeevan et al, 2003) several new tables and fields to existing tables were created to support the new functions added since.

For example, the **Geographic_Region** table to support the linking of a population to multiple geographic regions and the table ‘**Synonyms**’ to facilitate the new keyword search function and display of alternate names/synonyms for a locus, site or population. Two new fields for dbSNP accession number and chromosomal position were added to the ‘**Sites**’ table to permit the ordered display of sites under a selected locus by their chromosomal positions.

### The focus of ALFRED

The structure described above is designed for allele frequencies at nuclear DNA polymorphisms. Mitochondrial DNA (mt DNA) is uniparentally inherited (through females) and generally analyzed as a gene tree rather than as allele frequencies. The variation on the non-recombining part of the Y is similarly uniparently inherited (through males) and similarly analyzed. Both require a different database structure and are not considered in ALFRED.

### Sources of input data

Sources of data in ALFRED include the following:

- Data extracted from published literature. ALFRED researchers and curators routinely scan through the literature to find relevant papers containing allele frequency data of interest. The allele frequency data and related information from the selected papers are first extracted by the curatorial staff into an Excel Spread sheet. In the spreadsheet, the curators associate the extracted datasets with existing ALFRED data objects (eg, samples and sites). A software utility then reads the spreadsheet file, performs data integrity checks, and uploads the data into ALFRED.
- Data generated in the Kidd Lab are maintained in a separate database called PhenoDB (Cheung et al, 1996), which is accessible to laboratory members only. The data to be made public are automatically transferred from PhenoDB to ALFRED through a web application that is only accessible to ALFRED curators. This web application employs metadata and XML technologies to perform data translation between PhenoDB and ALFRED, as the databases were designed and developed independently to address different needs. The application maps heterogeneous but related data between the two databases.
- Data submitted by collaborators or other researchers in electronic format are first extracted into the standard Excel Spreadsheet and a number of curatorial steps as described above are performed before the data are uploaded into the database.

## User interface

The web user interface of ALFRED allows the users to easily query and display allele frequencies and related data. It has the following organization. Populations are organized by geographic regions and each population record is annotated with alternate-names (synonyms), linguistic, geographical location information and links to external databases such as Ethnologue and Rosetta Project for additional information. For example, see (http://alfred.med.yale.edu/alfred/recordinfo.asp?UNID=PO000036J). Population samples are organized by populations and annotated with sample information such as sample-size and relation to other samples. Loci are organized by chromosome and each locus record is annotated with alternate-names (synonyms), chromosomal position and a valid locus symbol and links to external databases such as GDB, Entrez Gene, UniGene. For example, see (http://alfred.med.yale.edu/alfred/recordinfo.asp?UNID=LO000422I). Genetic polymorphisms and haplotypes are organized by locus and each polymorphism record is annotated with dbSNP rs number (refSNP Identifier), alternate-names (synonyms), ancestral allele, and links to external databases for expanded molecular information such as dbSNP. For example, see (http://alfred.med.yale.edu/alfred/recordinfo.asp?UN ID=SI000188R). The active links to other databases provided from ALFRED’s populations, loci, and sites information pages facilitate easy retrieval of additional information. Each allele frequency record displayed is linked to the population sample information, polymorphism information, typing method and the publication the frequency was extracted from. Most publication entries are linked to PubMed for relevant citation.

Data queries can start with locus, population, publication author, ALFRED UID, dbSNP accession numbers (either refSNP accession number (rs#) or submitter-supplied accession number (ss#)), geographic region or a combination of gene name and population name. The results of frequency searches can be viewed both in graphical and tabular format. The graphical stacked-bar format offers a quick visual display of the frequency variation among populations (http://alfred.med.yale.edu/alfred/mvograph.asp?siteuid=SI001272M). On the other hand, the tabular format offers frequency values and related information which can be used in analysis tools (http://alfred.med.yale.edu/alfred/SiteTable1A_working.asp?siteuid=SI001272M).

### GIS-based map interface to display population data on a map

In population genetics, speciation events as well as more graded evolutionary changes over time in population structure are among the phenomena of interest. Interestingly, various geographical factors are among the identifiable and measurable parameters that are found to help understand genetic changes in populations over time. For instance, major isolating factors such as distance, deserts, mountain ranges, oceans, islands, and even latitude (tapping indirect factors such as climate, intensity of solar radiation exposure) help to identify and capture sources of variation in how modern human populations have evolved. The same factors would be relevant to the evolution of other species whether from the animal or plant kingdom. Clinal patterns of gene frequencies in existing populations sampled today can be signals that help us reconstruct ancient waves of human population migration. Some of the observed discontinuities in many different gene frequency patterns sampled from the genome may represent indications of past population formation/subdivision or of random genetic drift as a consequence of “founder” effects or population “bottlenecks”. These and many other related ideas have long been the subject of study and interest. For some recent examples from human population genetics of this very extensive and rich literature see Chikhi et al (1998), Reich and Goldstein (1998), Jorde et al (2000), and Rosser et al (2000).

The ability to visualize genetic data on human polymorphisms in a geographic context is an important aspect of interpreting the data, be they classical markers (Cavalli-Sforza et al, 1994), mtDNA data (eg, Torroni et al, 2001), markers on the non-recombining part of the Y (NRY) (eg, Rootsi et al, 2004), or autosomal DNA polymorphisms (eg, Tishkoff and Kidd, 2004). This has generally been done largely “by hand”, either literally or with graphic programs designed for the specific task. ALFRED has always displayed data, either tabularly or graphically, with populations grouped by general geographic region ordered roughly by distance from Africa but in largely arbitrary order within regions. A linear order, however, cannot adequately represent the two-dimensional surface of the earth. As the numbers of populations and autosomal markers in ALFRED have increased, a geographic display has become increasingly important.

Two aspects of the data benefit from a geographic display: selection of populations based on their location and display of data at the location of the population/sample. Several months ago we added a prototype geographic interface to display locations of populations and allow sets of populations to be selected. Unfortunately, making that interface compatible with multiple browsers was not possible. Moreover, the package was not readily amenable to display of data. Development was shifted to an interface that would interact with GIS (Geographic Information System) servers to allow much more flexibility and place ALFRED data into a standard format for correlation with extensive data accessible through GIS. The ALFRED Map Interface is now online (http://alfredgis.med.yale.edu/maps/?mode=None) and being systematically integrated into ALFRED.

**Figure 2:**
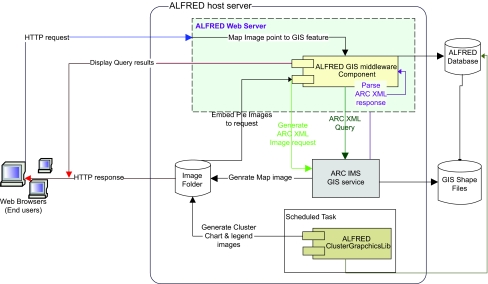
Architecture of map interface

**Figure 3:**
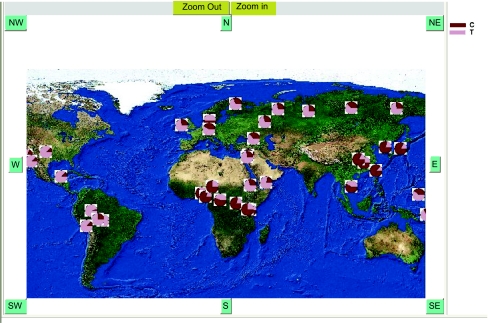
Pie chart displayed on the GIS Map - distribution of allele frequencies for the TaqMan (C__8829451_10, rs1229966)

This prototype web-based geographic map interface uses a GIS package called “ArcIMS” [Arc Internet Mapping Server]. The GIS web interface supports an integrated view that encompasses queries to ALFRED and GIS servers (Figure 2). The user can view populations and charts graphically on a map by filtering on population, sample, markers and also query detailed information from the ALFRED database by clicking on points and regions on the displayed map. The web interface seamlessly integrates with the GIS server through the ALFRED middleware component that primarily serves as a communication layer by generating XML requests and parsing the response from the GIS server. This component is also responsible for embedding pie chart images on the fly and mapping image points to GIS feature coordinates. This enables the web interface to visually depict allele frequencies across populations as Pie charts when the user selects a polymorphic site (Figure 3). This initial view displays a pie chart at the location of each population having that site. Chart images are pre-created by a graphing tool by taking the cumulative average of allele frequencies across samples for each population and site. The interface also supports a detailed view showing the frequencies for each sample. In this case the interface draws pie charts on demand in a popup when the user clicks on a pie chart that shows average allele frequencies across samples in the initial view. Information is also displayed about each sample accompanied by pie charts depicting the allele frequencies for the sites typed on the selected sample. In addition, links to allele frequency pie charts displayed on the map interface is provided from the polymorphism information page of ALFRED.

## Data output format

Users frequently want to analyze in various ways data retrieved from ALFRED. Several options are available. The existing HTML screen display of the allele frequency tables can be captured and imported into a spreadsheet. Though inadequate for statistical analysis, the stacked-bar graphics output can be captured as an image to illustrate variation. All the allele frequency tables (in semicolon-delimited format), polymorphismand population information tables (in tab-delimited format) can be downloaded in text format as well. In addition, the entire database can be downloaded in XML format by following the link provided in the web site (http://alfred.med.yale.edu/alfred/xmldatadump.asp). The XML format is more appropriate for exchanging data between different database applications.

## The empty matrix

Several search procedures to identify the availability of allele frequency tables for a particular polymorphism and population combination are accessible from the ALFRED home page. Several graphical overviews are also available to direct users to the more extensive ‘comparative’ aspects of the database. A ‘sites per population’ web page (http://alfred.med.yale.edu/alfred/sitesperpop_graphbycount.asp) shows graphically (and numerically) the number of allele frequency tables for each population. Currently, the maximum is 1033 for the Han. A ‘populations per site’ web page (http://alfred.med.yale.edu/alfred/popspersite_graphbycount.asp) represents the number of allele frequency tables for each polymorphic site. Currently, the Alu-insertion/deletion polymorphic sites and the forensic markers top the list with ACE Alu insertion having the maximum (frequency tables for 172 population samples). However, though some populations have data on many markers and some markers have data on many populations, the matrix of sites by populations is largely empty. Anthropologic genetics is plagued by this empty matrix problem: the paucity of markers uniformly typed on a large number of populations.

Preliminary analysis on data sets extracted from ALFRED led us to an important conclusion that there is no adequate data set in ALFRED to perform any comprehensive allele frequency data analysis exercise. There has not previously been such a large compendium of DNA polymorphism data on human polymorphisms that ALFRED now provides. (Classical markers are summarized in Cavalli-Sforza et al, 1994.) A survey of data assembled in ALFRED shows that only approximately two dozen markers have been uniformly studied on the same 100+ populations. Though simulation studies show that more than two dozen markers is needed for accurate estimation of fine-scale population relationships (eg, Kidd et al, 1974), solely by virtue of their having already been typed on multiple populations, these two dozen markers became a logical initial set for use in new populations to maximize global comparability. To get a discussion going, we have put together a suggested set of polymorphic sites based on what is already found in ALFRED and what we know about ongoing research. A list of the proposed sites along with a section where researcher’s comments can be submitted and posted can be found on the following ALFRED webpage: http://alfred.med.yale.edu/alfred/calling_researchers,asp. If researchers can come together to address this problem, detailed definitions of population relationships could be assessed both worldwide and regionally.

## Recent and future directions

Recent and future extensions of ALFRED would benefit the population genetics community. These extensions include the following:

### Synchronization of ALFRED polymorphism location information with NCBI’s mapping information

A function that has been recently implemented in the public interface is the synchronization of ALFRED polymorphism location information with NCBI’s genetic mapping information. When polymorphisms are displayed for the user, ALFRED is following the latest dbSNP build available from NCBI. This will help ALFRED users to browse and find markers of interest to them more efficiently.

### Polymorphism Markup Language (PML)

The ALFRED group has been involved in an international effort to standardize the XML representation of Single Nucleotide Polymorphism (SNP) data. This standardized XML syntax is called Polymorphism Markup Language (PML). In March of 2005, PML was accepted as an adopted specification by Object Management Group (OMG). The consortium includes representatives of major SNP-related databases including dbSNP, PharmGKB, HGVBase, JSNP, ALFRED, HapMap, and the Chinese Population Genetic Diversity database. ALFRED will be set up to export data in PML format in the near future.

### Database extensions

We will implement more data export formats (eg, PHYLIP) for supporting different types of evolutionary data analyses. We are in the process of extending ALFRED to provide genotype data for selected systems. We will also extend the database to model haplotype systems based on combination of multiple locus systems (sites). Such flexible modeling of haplotypes will facilitate the study of evolution.

### New User Interface Development

The current ALFRED public interface was designed to be a prototype. Expansion of ALFRED in quality and quantity is demanding a framework which is scalable, portable and facilitates easy integration with other software packages such as the GIS map interface. Apart from the flexibility that will be offered at the level of viewing and downloading of ALFRED data, several new functions will be incorporated into the database to enhance the quality and clarity of the displayed data.

### More flexible and efficient search and data download tools

ALFRED now offers several search tools for data searching and retrieval. Currently, ALFRED tables are available for download in text and XML format. But the increase in the contents in ALFRED and in the number of ALFRED users over the past 4 years requires more efficient and flexible search tools and download options. We are planning to consolidate all the different search tools ALFRED already offers and create new search tools that can retrieve and download data in an efficient manner. We are also planning to offer as download options various data formats that are being used by different population genetics programs. Though the matrix is still largely empty, there are subsets of populations and sites sufficient for meaningful analyses.

### Increased quantity and quality of data

We will continue to add more data to ALFRED as new data are published in the literature. In addition, we will explore the use of biomedical literature text mining tools to automate our data acquisition. For example, we have recently used PubMatrix (Becker et al, 2003) and PubCrawler (Hokamp and Wolfe, 2004) to facilitate search for allele frequency data in PubMed.

### Interoperability

We will implement a web service interface based on the Simple Object Access Protocol or SOAP (http://www.w3.org/TR/soap/) to provide a standard programmatic interface for external software applications to access the AFLRED database automatically. For example, an application can be written to construct phylogenetic trees based on allele frequency data by interoperating the ALFRED web service with the PHYLIP web service that is available at PathPort (Eckart and Sobral, 2003). As PML is becoming a standardized XML format for exchanging data between different SNP databases, we will develop applications to allow users to integrate data extracted from ALFRED with data extracted from other molecular-based SNP databases such as dbSNP and HapMap to allow more powerful evolutionary analyses. As ALFRED and other SNP databases evolve, PML will need to be changed. Plans are underway to develop a new version of PML (PML2) to accommodate such changes as well as new needs for SNP data analysis.

## Online Database References

ALFRED http://alfred.med.yale.edu

dbSNP http://ncbi.nih.gov/SNP

Ethnologue http://www.ethnologue.com

HapMap http://www.hapmap.org/

PathPort http://staff.vbi.vt.edu/pathport/services/

PubCrawler http://pubcrawler.gen.tcd.ie/

PubMatrix http://pubmatrix.grc.nia.nih.gov/
